# The nonlinear correlation between the cardiometabolic index and the risk of diabetes: A retrospective Japanese cohort study

**DOI:** 10.3389/fendo.2023.1120277

**Published:** 2023-02-16

**Authors:** Fubing Zha, Changchun Cao, Mengru Hong, Huili Hou, Qionghua Zhang, Bin Tang, Haofei Hu, Yong Han, Yibing Zan, Yulong Wang, Jianwen Xu

**Affiliations:** ^1^ Department of Rehabilitation Medicine, The First Affiliated Hospital of Guangxi Medical University, Nanning, Guangxi, China; ^2^ Department of Rehabilitation, Shenzhen Second People’s Hospital, The First Affiliated Hospital of Shenzhen University, Shenzhen, Guangdong, China; ^3^ Department of Rehabilitation, Shenzhen Dapeng New District Nan’ao People’s Hospital, Shenzhen, Guangdong, China; ^4^ Department of Nephrology, Shenzhen Second People’s Hospital, The First Affiliated Hospital of Shenzhen University, Shenzhen, Guangdong, China; ^5^ Department of Emergency, Shenzhen Second People’s Hospital, The First Affiliated Hospital of Shenzhen University, Shenzhen, Guangdong, China

**Keywords:** cardiometabolic index, triglyceride, high-density lipoprotein cholesterol, waist-to-height ratio, diabetes mellitus, non-linear

## Abstract

**Background:**

The cardiometabolic index (CMI) has been proposed as a novel indicator of cardiometabolic status. However, evidence on the relationship between CMI and diabetes mellitus (DM) risk was limited. Our study aimed to explore the relationship between CMI and DM risk among a large cohort of Japanese adults.

**Methods:**

This retrospective cohort study recruited 15453 Japanese adults without diabetes at baseline who underwent physical examinations at the Murakami Memorial Hospital between 2004 and 2015. Cox proportional-hazards regression was applied to evaluate the independent relationship between CMI and diabetes. Our study performed a generalized smooth curve fitting (penalized spline technique) and an additive model (GAM) to determine the non-linear relationship between CMI and DM risk. In addition, a set of sensitivity analyses and subgroup analyses were employed to evaluate the relationship between CMI and incident DM.

**Results:**

After adjusting for confounding covariates, CMI was positively related to the DM risk in Japanese adults (HR: 1.65, 95%CI: 1.43-1.90, P<0.0001). A series of sensitivity analyses were also employed in this study to guarantee the reliability of the findings. In addition, our study discovered a non-linear association between CMI and diabetes risk. CMI’s inflection point was 1.01. A strong positive association between CMI and diabetes incidence was also discovered to the left of the inflection point (HR: 2.96, 95%CI: 1.96-4.46, P<<0.0001). However, their association was not significant when CMI was higher than 1.01 (HR: 1.27, 95%CI: 0.98-1.64, P=0.0702). Interaction analysis showed that gender, BMI, habit of exercise, and smoking status interacted with CMI.

**Conclusion:**

Increased CMI level at baseline is associated with incident DM. The association between CMI and incident DM is also non-linear. A high CMI level is associated with an increased risk for DM when CMI is below 1.01.

## Introduction

Diabetes Mellitus (DM) is a metabolic disorder with chronic hyperglycemia. According to the International Diabetes Federation’s epidemiological data, approximately 463 million people aged 20-79 years were diagnosed with diabetes worldwide in 2019, with a prevalence of 9.3% (1). Diabetes is one of the most common metabolic diseases and imposes a heavy economic burden on patients and their countries (2). Although DM is irreversible, it is mainly preventable (3). In order to prevent and detect DM, it is crucial to completely comprehend the risk factors for the disease.

The distribution of body fat accumulation significantly impacts the onset of metabolic syndrome, diabetes, and insulin resistance (IR) (4, 5). Previous research found a strong association between type 2 diabetes mellitus (T2DM) and a variety of traditional obesity indicators, including waist circumference (WC) and body mass index (BMI). Additionally, it has been proposed that the waist-to-height ratio (WHtR) is more accurate than BMI and WC for identifying cardiovascular risk, including T2DM (6, 7). The triglycerides to high-density lipoprotein cholesterol ratio (TG/HDL-C ratio), which has been shown to affect type 2 diabetes, is another reliable and simple insulin resistance measurement (8–10). Wakabayashi I et al. (11) first developed the cardiometabolic index (CMI), which was the product of WHtR and TG/HDL-C and could be used to determine the risk of cardiometabolic disease and type 2 diabetes (12–14). CMI is an excellent predictor of type 2 diabetes compared to other obesity and lipid indicators, such as BMI, body mass index (BAI), WC, and triglycerides (TG) (11, 15, 16). Furthermore, CMI is connected to some obesity-related metabolic disorders, including hyperuricemia, nonalcoholic fatty liver disease, renal disease, and stroke (17–20). Most recent studies on the relationship between CMI and diabetes have cross-sectional designs and small sample numbers. Unfortunately, neither the non-linear relationship between CMI and DM nor subgroup analyses were performed. Therefore, a retrospective cohort study was designed to observe the relationship between CMI and DM in a sizable cohort of Japanese adults. Furthermore, this study directed therapeutic practice by exploring the quantitative association between CMI and DM in different genders.

## Methods

### Data source

The information was derived from this study: Takuro Okamura et al. (21): Ectopic fat obesity presents the greatest risk for incident type 2 diabetes: a population-based longitudinal study. Dryad Digital Repository (https://doi.org/10.1038/s41366-018- 0076-3). Under the Dryad terms of service, researchers can use the data for secondary analysis without harming the authors following the Dryad terms of service.

### Study participants

Written informed consent was obtained from each participant in the initial study, which was carried out with the Murakami Memorial Hospital’s Clinical Research Ethics Committee (21). Therefore, this secondary analysis did not need ethical approval. Additionally, the initial study was conducted under the Declaration of Helsinki. All procedures, including the declarations in the Declarations section, were carried out under the relevant norms and laws.

**Figure 1:**
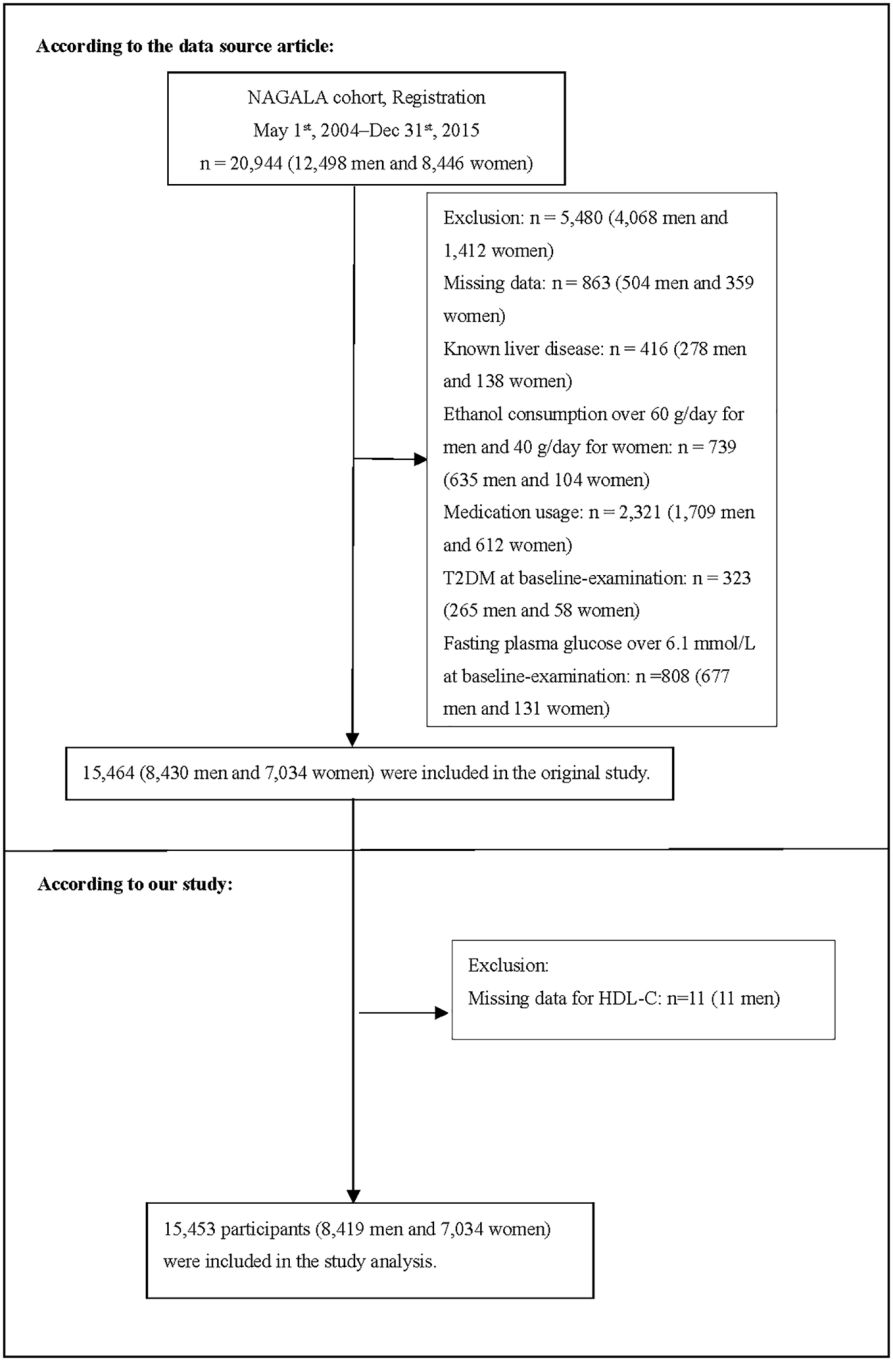
Study Population.

The original study initially enrolled 20944 Japanese individuals who took the physical exam between 2004 and 2015 and completed at least a second exam. Afterward, 5491 individuals were excluded, and 15453 individuals (8419 male and 7034 female) were left for data analysis of our study (**Figure 1**). If subjects met any of the following criteria at the baseline, they were excluded from the study: (1) type 2 diabetes; (2) known liver disease (such as hepatitis B or hepatitis C at baseline); (3) alcoholic fatty liver disease; (4) fasting plasma glucose (FPG)≥ 6.1 mmol/L; (5) missing data of variables; (6) incomplete HDL-C; (7) taking any medication.

### Covariates

In our research, covariates were chosen following our clinical expertise and prior research. The following variables were utilized as covariates: (1) categorical variables: smoking status, habit of exercise, and gender; (2) continuous variables: ethanol consumption, diastolic blood pressure (DBP), systolic blood pressure (SBP), age, BMI, alanine aminotransferase (ALT), gamma-glutamyl transferase (GGT), aspartate aminotransferase (AST), total cholesterol (TC), glycosylated hemoglobin (HbA1c), and FPG. Through the use of a standardized self-management questionnaire, information on each individual’s medical history and lifestyle characteristics were gathered for the original study. The professional staff accurately measured the subject’s height, weight, WC, and blood pressure. The original study team used a consistent process to acquire laboratory test data under controlled circumstances. The initial research evaluated ethanol consumption based on subjects’ ethanol consumption during the prior month and then calculated the average weekly ethanol consumption (21).

### Cardiometabolic index

CMI is regarded as a continuous variable. [WC (cm)/height (cm)]×[TG (mmol/L)/HDL-C (mmol/L)] was the formula used to calculate CMI in detail (11).

### Diagnosis of incident diabetes

Diabetes was defined as glycosylated hemoglobin ≥ 6.5%, fasting plasma glucose ≥ 7 mmol/L (22), or self-reported during the follow-up period.

### Statistical analysis

Statistical analysis was employed using the R software package (version 3.3.1) (www.r-project.org, The R Foundation) and Empower-Stats (version 2.16.1) (www.empowerstats.com, XY Solutions, Inc., Boston, MA).

We explored the characteristics of all subjects at baseline according to quartiles of CMI. Skewed and normally distributed continuous variables were described as median (quartile) and mean ± standard deviation, respectively. The differences among the CMI groups were compared by one-way ANOVA test, Kruskal-Wallis H test, or chi-square test. Comparisons of survival and cumulative event rates were conducted using the Kaplan-Meier method. In addition, we compared the Kaplan-Meier hazard ratios (HR) of adverse events by log-rank test (23).

The multivariate Cox regression analysis was also used to explore the association of CMI with the risk of DM. Furthermore, we constructed three models to assess the association between CMI and diabetes risk: crude Model, Model I, and Model II. We only adjusted for these covariances if the hazard ratios changed by ≥10% when added to the adjusted Model (24).

Since CMI was a continuous variable, we tried to identify the nonlinear relationship between CMI and diabetes through Cox proportional hazards regression model with cubic spline functions and the smooth curve fitting (penalized spline method). If the relationship was nonlinear, we employed the two-piece linear regression model to determine the inflection point (25). The present study used the log-likelihood ratio to describe the most appropriate Model for the association of CMI with diabetes.

The present study employed a series of sensitivity analyses to assess robust findings. We converted CMI into a categorical variable according to the quartile. Then we calculated P for the trend to verify the results of CMI as the continuous variable and examine the possibility of nonlinearity. Obesity and the elderly were associated with an increased risk of diabetes. Hence, we excluded individuals with BMI≥25kg/m^2^ or age≥60 years in other sensitivity analyses to assess the relationship between CMI and diabetes risk. Furthermore, the present study employed a generalized additive model (GAM) to incorporate the continuity variables into the equation as a curve to examine the robustness of our findings. To assess the impact of potential unmeasured confounding between CMI and the risk of diabetes, we further calculated E-values (26).

Moreover, we applied the Cox proportional hazard model to the subgroup analysis (ethanol consumption, habit of exercise, smoking status, BMI, age, and gender). Firstly, the interaction test between these variables and CMI was performed before the subgroup analysis. The likelihood ratio test was used to compare models with and without the multiplicative interaction term. Secondly, continuous variables, including BMI and age, were converted into categorical variables based on clinical cut-off points age (<60, ≥60 years) and BMI (<25, ≥25kg/m^2^). Thirdly, a fully adjusted analysis was performed for each stratum, except for the stratification factor. Ultimately, the likelihood ratio test was used to determine whether interaction terms existed in models with and without interaction terms. STROBE statement was obeyed during the whole research (24, 27). Statistical significance was determined by P < 0.05 in two-tailed tests.

## Results

### Characteristics of participants

**Table 1** presented all eligible individuals’ basic clinical measurements, biochemical tests, and other parameters. The final analysis included 15453 individuals, with a mean age of 43.71 ± 8.90 years and a male participation rate of 54.48%. 373 participants eventually got diabetes after a median of 5.39 years of follow-up. The mean ± SD of WC, BMI, TG, HDL-C, and CMI were 76.47 ± 9.11 cm, 22.12 ± 3.13 kg/m^2^, 0.91 ± 0.66 mmol/L, 1.46 ± 0.40 mmol/L and 0.36 ± 0.39. We assigned participants into subgroups using CMI quartiles (≤0.133, 0.133-0.230, 0.230-0.423, > 0.423). In the highest CMI group, individuals had higher ethanol consumption, DBP, SBP, WC, BMI, age, ALT, AST, GGT, TG, TC, HbA1c, FPG, higher rates of men, smokers, but lower HDL-C and lower rates of the habit of exercise.

**Table 1:** The Baseline Characteristics of participants.

CMI	Q1 (≤0.133)	Q2 (0.133 to ≤0.233)	Q3 (0.233 to ≤0.437)	Q4 (>0.437)	P-value
**Participants**	3863	3863	3863	3864	
**Gender**					<0.001
Female	2951 (76.39%)	2136 (55.29%)	1330 (34.43%)	617 (15.97%)	
Male	912 (23.61%)	1727 (44.71%)	2533 (65.57%)	3247 (84.03%)	
**Age(years)**	40.98 ± 8.27	43.23 ± 8.82	45.10 ± 9.05	45.53 ± 8.69	<0.001
**Ethanol consumption(g/week)**	1 (0, 22)	1 (0, 60)	2.8 (0, 84)	12(1, 90)	<0.001
**Smoking status**					<0.001
Never-smoker	3029 (78.41%)	2513 (65.05%)	1980 (51.26%)	1505 (38.95%)	
Ex-smoker	465 (12.04%)	671 (17.37%)	849 (21.98%)	964 (24.95%)	
Current-smoker	369 (9.55%)	679 (17.58%)	1034 (26.77%)	1395 (36.10%)	
**Habit of exercise**					<0.001
No	3125 (80.90%)	3176 (82.22%)	3155 (81.67%)	3291 (85.17%)	
Yes	738 (19.10%)	687 (17.78%)	708 (18.33%)	573 (14.83%)	
**SBP (mmHg)**	107.67 ± 12.81	112.10 ± 13.89	116.36 ± 14.46	121.84 ± 14.86	<0.001
**DBP (mmHg)**	66.55 ± 9.06	69.76 ± 9.74	72.98 ± 9.95	77.03 ± 10.26	<0.001
**BMI (kg/m^2^)**	19.98 ± 2.13	21.27 ± 2.47	22.62 ± 2.70	24.59 ± 3.08	<0.001
**WC (cm)**	69.54 ± 6.46	73.83 ± 7.37	78.26 ± 7.48	84.24 ± 7.82	<0.001
**ALT (IU/L)**	14 (11, 17)	15 (12, 19)	18 (14, 23)	23 (17, 33)	<0.001
**AST (IU/L)**	16 (13, 19)	17 (14, 20)	17 (14, 21)	19 (16,24)	<0.001
**GGT(IU/L)**	12 (10, 15)	13 (11, 18)	16 (12, 23)	22 (16, 34)	
**HDL-C (mmol/L)**	1.84 ± 0.38	1.57 ± 0.29	1.35 ± 0.25	1.09 ± 0.21	<0.001
**TG (mmol/L)**	0.38 (0.30, 0.46)	0.61 (0.52, 0.71)	0.88 (0.76, 1.03)	1.52 (1.24, 1.95)	<0.001
**TC (mmol/L)**	4.85 ± 0.80	5.00 ± 0.81	5.18 ± 0.85	5.47 ± 0.87	<0.001
**HbA1c (%)**	5.13 ± 0.30	5.15 ± 0.31	5.18 ± 0.33	5.22 ± 0.34	<0.001
**FPG (mmol/L)**	4.96 ± 0.39	5.10 ± 0.39	5.22 ± 0.39	5.35 ± 0.37	<0.001
**CMI**	0.09 (0.07, 0.11)	0.18 (0.15, 0.20)	0.31 (0.27, 0.37)	0.68 (0.53, 0.95)	<0.001

Values are n(%) or mean ± SD or median (quartile).

CMI, cardiometabolic index; BMI, body mass index; WC, waist circumference; SBP, systolic blood pressure; DBP, diastolic blood pressure; ALT, alanine aminotransferase; AST, aspartate aminotransferase; GGT, gamma-glutamyl transferase; HDL-C, high-density lipoprotein cholesterol; TC, total cholesterol; TG, triglycerides; HbA1c, hemoglobin A1c; FPG, fasting plasma glucose.

### The results of the relationship between CMI and incident diabetes

**Table 2** revealed that 373 participants developed diabetes. The total incidence rate of all persons was 399.14 per 100,000 person-years. In particular, the incidence rate of the four CMI groups were 103.16, 160.79, 331.58, and 956.27 100,000 person-years, respectively. Participants with a high CMI level had higher incidence rates of diabetes than those with the lowest CMI level (P < 0.0001 for trend).

**Table 2:** Relationship between CMI and incident diabetes in different models.

CMI	Participants (n)	DM events (n)	Incidence rate (per 100,000 person-year)	Crude Model (HR, 95% CI, P)	Model I (HR, 95% CI, P)	Model II (HR, 95% CI, P)	Model III (HR, 95% CI, P)	Model IV (HR, 95% CI, P)	Model V (HR, 95% CI, P)
Total	15453	373	399.14	2.22 (2.05, 2.41) <0.0001	1.98 (1.78, 2.19) <0.0001	1.65 (1.43, 1.90) <0.0001	1.59 (1.37, 1.84) <0.0001	1.50 (1.18, 1.90) 0.0010	1.65 (1.42, 1.91) <0.0001
Q1	3863	103.16	103.16	ref	ref	ref	ref	ref	ref
Q2	3863	160.79	160.79	1.48 (0.88, 2.51) 0.1403	1.17 (0.69, 1.99) 0.5711	0.92 (0.54, 1.58) 0.7747	0.91 (0.53, 1.56) 0.7276	0.80 (0.45, 1.43) 0.4594	1.09 (0.62, 1.92) 0.7683
Q3	3863	331.58	331.58	3.04 (1.90, 4.87) <0.0001	1.99 (1.22, 3.25) 0.0062	1.23 (0.75, 2.02) 0.4065	1.15 (0.69, 1.91) 0.5902	1.12 (0.65, 1.93) 0.6736	1.27 (0.75, 2.17) 0.3714
Q4	3864	956.27	956.27	8.70 (5.62, 13.48) <0.0001	4.79 (2.97, 7.74) <0.0001	2.04 (1.26, 3.32) 0.0040	1.86 (1.13, 3.07) 0.0153	1.40 (0.81, 2.44) 0.2323	2.18 (1.30, 3.68) 0.0034
P for trend			<0.0001	<0.0001	<0.0001	<0.0001	0.0003	0.0334	<0.0001

Crude Model: we did not adjust for other covariants.

Model I: we adjusted for gender, age, ethanol consumption, smoking status, habit of exercise, SBP, and DBP.

Model II: we adjusted for gender, age, ethanol consumption, smoking status, habit of exercise, SBP, DBP, ALT, AST, GGT, TC, HbA1c, and FPG.

Model III: we adjusted for gender, age, ethanol consumption, smoking status, habit of exercise, SBP, DBP, ALT, AST, GGT, TC, HbA1c, and FPG. However, continuous covariates were adjusted as nonlinearity.

Model IV: was sensitivity analysis after excluding those with BMI≥25kg/m^2^. We adjusted gender, age, ethanol consumption, smoking status, habit of exercise, SBP, DBP, ALT, AST, GGT, TC, HbA1c, and FPG.

Model V: was sensitivity analysis after excluding those with age≥60 years. We adjusted gender, ethanol consumption, smoking status, habit of exercise, SBP, DBP, ALT, AST, GGT, TC, HbA1c, and FPG.

HR, hazard ratio; CI, confidence interval; Ref, Reference; CMI, cardiometabolic index.

**Figure 2:**
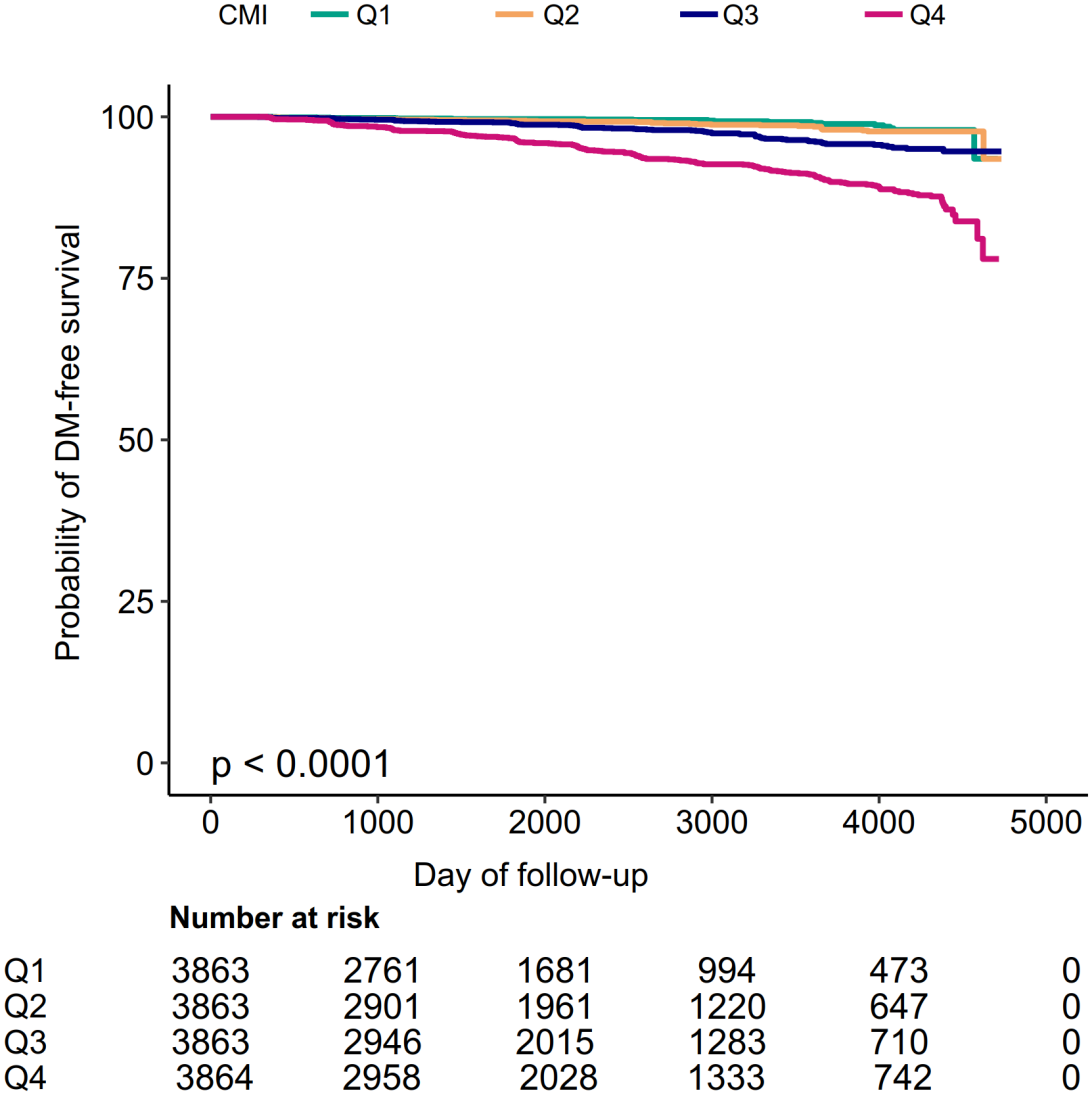
Kaplan–Meier event-free survival curve. Kaplan–Meier event-free survival curve. Kaplan–Meier analysis of incident diabetes based on CMI quartiles (log-rank, P < 0.0001).

Kaplan-Meier curves for the probability of diabetes-free survival were depicted in **Figure 2**. A significant difference existed between the four CMI groups regarding diabetes risk (P<0.0001). There was a gradual decrease in the probability of diabetes-free survival as CMI levels increased. Therefore, individuals in the top CMI group had the highest risk of diabetes.

**Table 2** showed the Cox proportional hazard regression models, which assessed the association between CMI and diabetes risk. Both the three adjusted and unadjusted models were presented in **Table 2**. In the crude mode, CMI was positively correlated with diabetes (HR: 2.22, 95%CI: 2.05-2.41, P<0.0001). In Model I (adjusting for gender, ethanol consumption, habit of exercise, smoking status, age, DBP, and SBP), the findings did not have apparent changes (HR: 1.98, 95%CI: 1.78-2.19, P<0.0001). In Model II (adjusting for gender, ethanol consumption, habit of exercise, smoking status, age, DBP, SBP, AST, GGT, ALT, TC, FPG, and HbA1c), the risk of DM increased by 65% for each unit increase in CMI (HR: 1.65, 95%CI: 1.43-1.90, P<0.0001).

### Sensitive analysis

To examine the robustness of our conclusions, we employed a series of sensitivity analyses. CMI was transformed into a categorical variable (based on quartile) and reinserted into the models. Compared with the Q1 group, the adjusted HR (95% CI) for the Q4 group was 2.04 (1.26-3.32). Furthermore, when CMI was transformed into a categorical variable, the trends of HR were not equal, suggesting a possible nonlinear association between CMI and diabetes risk. Additionally, the continuity covariate was inserted into the equation by a GAM. The findings of Model III were consistent with the results of Model II (HR: 1.59, 95%CI:1.37-1.84, P<0.0001) (**Table 2**). Besides, to evaluate the impact of potential unmeasured confounding between CMI and the risk of diabetes, this study further generated E-values. The E value for this study was 2.69 (95%CI: 2.65-2.72). Compared to the relative risk and CMI for unmeasured confounding variables, the E value for this study was larger. The results indicated that unknown or unmeasured confounding variables hardly affected the association between CMI and the risk of diabetes.

In addition, individuals with a BMI ≥ 25kg/m^2^ were excluded from other sensitivity analyses. In Model IV, after adjusting gender, ethanol consumption, habit of exercise, smoking status, age, DBP, and SBP, AST, GGT, ALT, TC, FPG, HbA1c, there was also a positive association between CMI and diabetes risk (HR: 1.50, 95%CI: 1.18-1.90) (**Table 2**). Individuals with age ≥ 60 years were also excluded from other sensitivity analyses. The results suggested that after adjusting for gender, ethanol consumption, habit of exercise, smoking status, DBP, SBP, AST, GGT, ALT, TC, FPG, and HbA1c, CMI was still positively correlated with diabetes risk (HR: 1.65, 95%CI: 1.42-1.91) in Model V (**Table 2**). Based on the sensitivity analyses, our findings were well-robust.

### The analyses of the non-linear relationship

**Figure 3:**
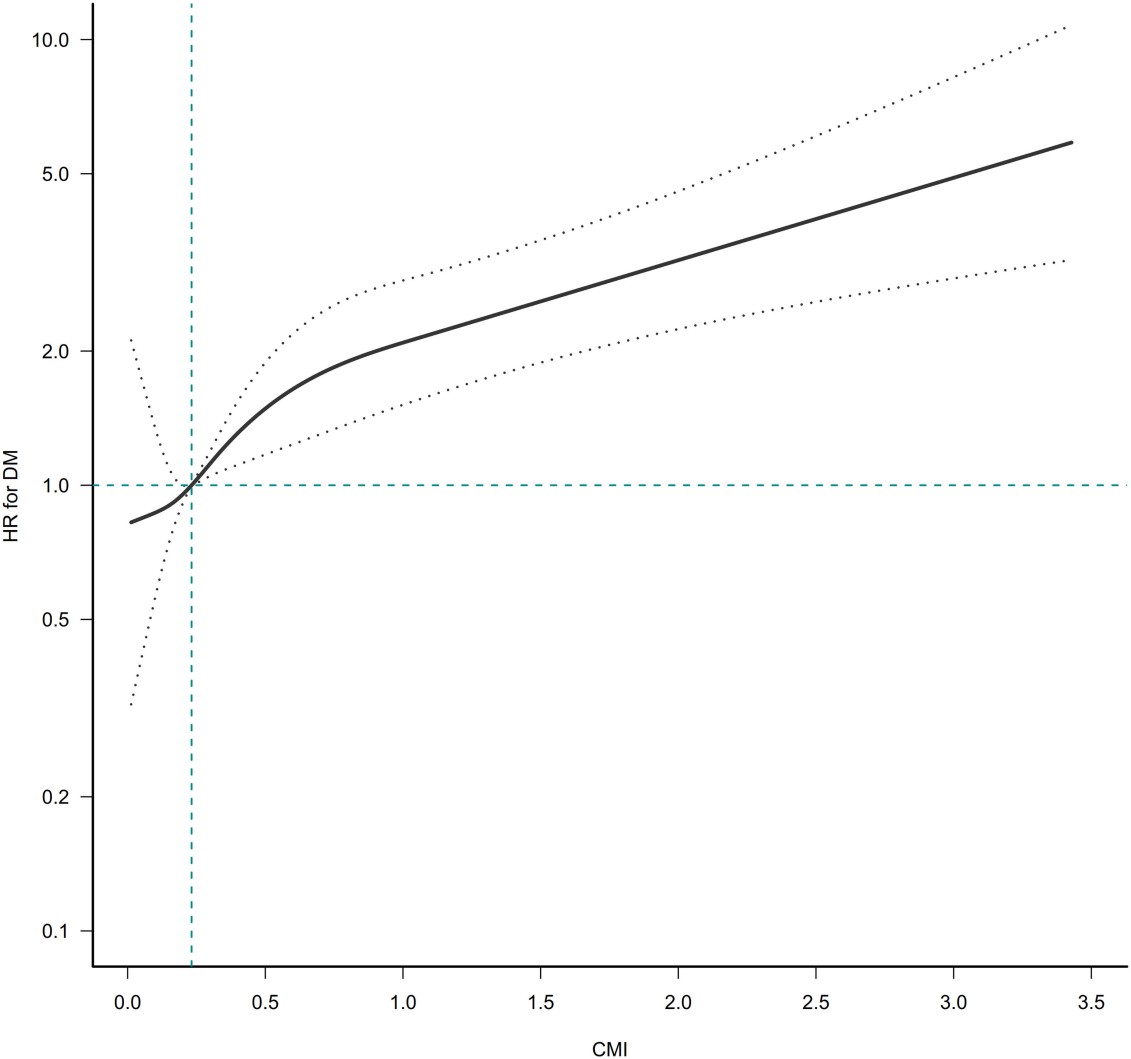
The nonlinear relationship between CMI ratio and incident diabetes. A nonlinear relationship was detected after adjusting for gender, age, ethanol consumption, smoking status, habit of exercise, SBP, DBP, ALT, AST, GGT, TC, HbA1c, and FPG.

The GAM and the smooth curve fitting (penalty curve method) were applied to verify the nonlinearity in the association between CMI and the risk of diabetes (**Figure 3**). **Table S1** revealed a nonlinear association between CMI and diabetes after adjusting for gender, ethanol consumption, habit of exercise, smoking status, age, DBP, SBP, AST, GGT, ALT, TC, FPG, and HbA1c. According to a two-piecewise linear regression model, the present study observed the inflection point of CMI was 1.01 (P for the log-likelihood ratio test= 0.003). When CMI was lower than 1.01, CMI was positively associated with diabetes risk (HR: 2.96, 95%CI: 1.96-4.46, P<<0.0001). In contrast, when CMI was above 1.01, their association was not significant (HR: 1.27, 95%CI: 0.98-1.64, P=0.0702).

### The results of the subgroup analysis

**Figure 4:**
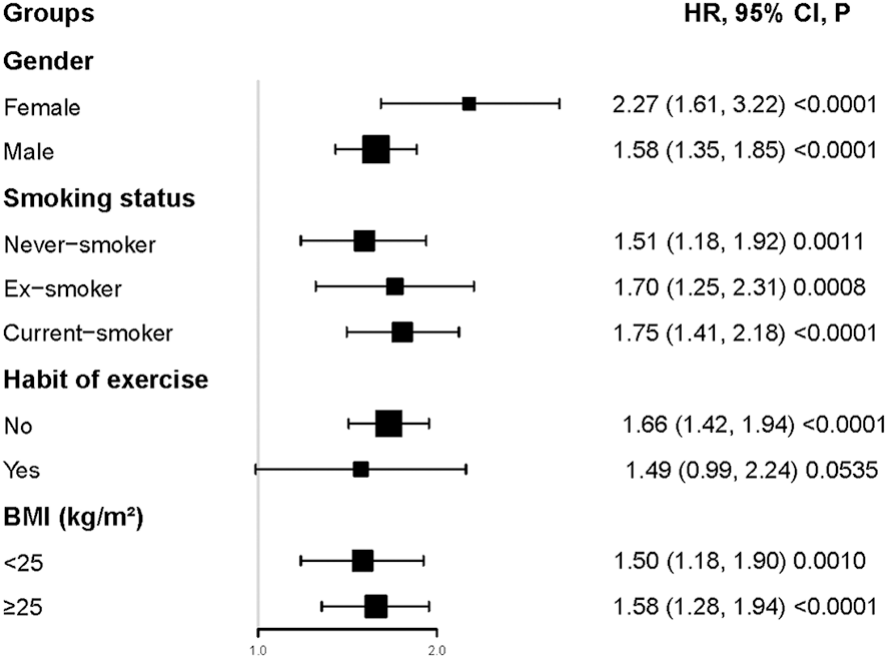
Subgroups analysis. Specifically, a stronger relationship between CMI and diabetes risk was observed in participants with female, current-smoker, lack of exercise habits, and BMI≥25kg/m^2^. In contrast, a weaker relationship between CMI and diabetes risk was observed in participants with male, habit of exercise, never-smoker, ex-smoker, and BMI< 25kg/m^2^.

Interaction tests performed before subgroup analyses showed that gender, BMI, habit of exercise, and smoking status interacted with CMI (P<0.001). In contrast, age and ethanol consumption did not interact with CMI (P>0.05) (**Table S2**). Therefore, further subgroup analyses with gender, BMI, habit of exercise, and smoking status were performed (**Figure 4**). Specifically, a stronger relationship between CMI and diabetes risk was observed in participants with female, current-smoker, lack of exercise habits, and BMI ≥ 25kg/m^2^. In contrast, a weaker relationship between CMI and diabetes risk was observed in participants with male, habit of exercise, never-smoker, ex-smoker, and BMI < 25kg/m^2^.

## Discussion

Our retrospective study investigated the association between CMI and the risk of developing diabetes in Japanese individuals. This study showed that higher CMI was associated with a higher risk of diabetes. The association between CMI on diabetes was also examined on the left and right sides of the inflection point. CMI level had a nonlinear association with incident diabetes. When CMI was below 1.01, we discovered a significant positive correlation between CMI and diabetes incidence (HR: 2.96, 95%CI: 1.96-4.46, P<0.0001). However, their association was not significant when CMI was higher than 1.01 (HR: 1.27, 95%CI: 0.98-1.64, P=0.0702). Interaction analysis showed that gender, BMI, habit of exercise, and smoking status interacted with CMI.

CMI is a newly proposed indicator related to cardiovascular risk factors, confirming its utility in the early detection of associated cardiovascular disorders (12, 14, 28). CMI could also identify the status of DM more accurately (11). CMI should be considered a combination of dyslipidemia and obesity, because it combines TG/HDL-C and WHtR and can be used as a valid differential indicator for diabetes. Our results are consistent with those of the following studies. In a cross-sectional study involving 11478 individuals in rural Northeast China, Shi WR et al. (29) demonstrated an association between elevated CMI and risk of developing diabetes after adjusting for race, age, income level, education level, medication usage, vegetable intake, meat intake, fatty food after intake, physical activity, family history of DM, hypertension, history of cardiovascular diseases, drinking status, and current smoking. In another cross-sectional study of 10196 subjects, Wakabayashi I et al. (11) discovered that after adjusting for age and histories of regular exercise, alcohol drinking, and smoking, there was a stronger association between CMI and diabetes. A longitudinal study of 7347 middle-aged and elderly Chinese showed that individuals with high CMI had a far higher risk of developing type 2 diabetes after adjusting for region, sex, age, marital status, ln(per capita expenditures), education, hypertension, drinking, smoking, low-density lipoprotein cholesterol (LDL-C), and TC (13). This study involved 15453 Japanese adults, and we found that the incidence of diabetes was higher with increased CMI levels. After adjusting for gender, ethanol consumption, habit of exercise, smoking status, age, DBP, SBP, AST, GGT, ALT, TC, FPG, and HbA1c, the results showed that the risk of DM increased by 65% for each unit increase in CMI. Furthermore, the sensitivity studies showed that this association could still be observed in Japanese adults with BMI < 25 kg/m^2^ or age < 60 years. Our study included a larger population compared to those of previous studies. In addition, we adjusted for more covariates, such as SBP, DBP, GGT, FPG, and HbA1c, which were all critical risk factors for diabetes. More importantly, we used sensitivity and subgroup analysis methods to validate further the solidity of the association between CMI and diabetes. In short, our results further confirmed the positive association between CMI and diabetes risk in the Japanese population. This study provided supporting evidence for clinical interventions for CMI levels to reduce the risk of diabetes.

To our knowledge, previous studies have not explored a possible curvilinear relationship between CMI and DM. The nonlinear association between CMI and DM in various genders was first examined in the current study. After adjusting for gender, ethanol consumption, habit of exercise, smoking status, age, DBP, SBP, AST, GGT, ALT, TC, FPG, and HbA1c, the smooth curve result revealed that the association between CMI and DM was nonlinear. We determined the CMI inflection point using a two-piecewise linear regression model. When the CMI level was lower than 1.01, the risk of DM increased by 196% for each unit increase in CMI (HR: 2.96, 95%CI: 1.96-4.46, P<<0.0001). CMI was not associated with incident DM when CMI was higher than 1.01 (HR: 1.27, 95%CI: 0.98-1.64, P=0.0702). Elevated CMI will indicate that participants have an increased risk of developing diabetes during follow-up, alerting people to make early changes in lifestyle habits to improve outcomes.

The mechanism by which CMI leads to the development of diabetes remains unclear. The aberrant lipid metabolism may account for these results in individuals with assessed CMI. In obese individuals with high WHtR, excess free fatty acids can impair insulin’s ability to play a role in glucose metabolism and lead to insulin resistance (30). In individuals with abdominal obesity, the number of insulin receptors on target tissues and the binding affinity decrease, resulting in a reduced ability to process glucose (31, 32). Meanwhile, elevated TG status contributes to the development of diabetes in a manner similar to abdominal obesity. It could be viewed as a crucial transitional stage from obesity to diabetes mellitus. Reduced HDL-C levels may negatively affect β cells’ ability, decreasing insulin sensitivity and output (32, 33).

Several strengths of our study can be found. First, we examined the nonlinear relationship using a GAM and a smooth curve fitting to identify the optimal inflection point for the effect of CMI on diabetes. Second, results were also rigorously adjusted statistically to reduce the influence of confounding factors, ensuring our findings’ reliability. Third, our results were tested for robustness through sensitivity analyses (CMI transformation, employing a GAM to put the continuity covariate into the equation as a curve, employing a GAM to put the continuity covariate into the equation as a curve, estimating E-values to examine the potential for unmeasured confounders, subgroup analysis, and reassessing the relationship between CMI on diabetes after excluding participants with BMI ≥ 25kg/m^2^ or age ≥ 60 years) to ensure their reliability. Fourth, we employed a subgroup analysis to find other risk factors that might affect the relationship between CMI and diabetes.

There are still some limitations in our study. First, our study includes only the Japanese population. As a result, other geographic and racial groups cannot use the study’s findings. Second, because individuals with excessive drinking habits, viral hepatitis, or drug use were excluded from the study, our findings may not apply to the broader population. In the future, we can consider designing our research or cooperation with other researchers to collect as many people as possible, including lack of data, excessive drinking habits, viral hepatitis, or drug use. Third, similar to the characteristics of all retrospective studies, our study may have had unmeasured or uncontrolled confounding covariates such as education, income, marital status, dietary factors and family history of diabetes. However, E values were calculated to quantify the potential impact of unmeasured confounding covariates. Unmeasured confounding covariates were unlikely to influence the relationship between CMI and the risk of diabetes. Fourth, the initial study only measured baseline WHtR, HDL-C, and TG. In addition, the initial investigation did not address changes in WHtR, HDL-C, and TG over time. In the future, we will consider designing our study to document more confounding factors, including education, income, marital status, dietary factors, family history of diabetes, and fluctuations in TG, WHtR, and HDL-C during follow-up. Therefore, we could explore the impact of changes in CMI on future diabetes risk through a GAM model.

## Conclusion

This cohort study shows a non-linear association between CMI and diabetes in the Japanese population. There is a strong positive association between CMI and the risk of developing diabetes when CMI is less than 1.01. These data provide strong evidence to enhance the value of CMI in evaluating diabetes.

## Data availability statement

The datasets presented in this study can be found in online repositories. The names of the repository/repositories and accession number(s) can be found below: https://datadryad.org/stash/dataset/doi:10.5061%2Fdryad.8q0p192.

## Ethics statement

The studies involving human participants were reviewed and approved by the Institutional Review Board of the Murakami Memorial Hospital. The patients/participants provided their written informed consent to participate in this study.

## Author contributions

FZ and CC contributed to the study concept and design, researched and interpreted the data, and drafted the manuscript. MH, HLH, and QZ examined the data and reviewed the manuscript. BT, HFH, YH, and YZ oversaw the project’s progress, contributed to the discussion and reviewed the manuscript. YW and JX are the guarantors of this work and, as such, had full access to all the data in the study and took responsibility for the integrity of the data and the accuracy of the data analysis. All authors contributed to the article and approved the submitted version.

## References

[1] Saeedi P Petersohn I Salpea P Malanda B Karuranga S Unwin N. Global and regional diabetes prevalence estimates for 2019 and projections for 2030 and 2045: Results from the international diabetes federation diabetes atlas, 9(th) edition. Diabetes Res Clin Pract (2019) 157:107843. doi: 10.1016/j.diabres.2019.107843 31518657

[2] Lin X Xu Y Pan X Xu J Ding Y Sun X. Global, regional, and national burden and trend of diabetes in 195 countries and territories: An analysis from 1990 to 2025. Sci Rep (2020) 10(1):14790. doi: 10.1038/s41598-020-71908-9 32901098 PMC7478957

[3] Wu Y Hu H Cai J Chen R Zuo X Cheng H. A prediction nomogram for the 3-year risk of incident diabetes among Chinese adults. Sci Rep (2020) 10(1):21716. doi: 10.1038/s41598-020-78716-1 33303841 PMC7729957

[4] Wang K Gong M Xie S Zhang M Zheng H Zhao X. Nomogram prediction for the 3-year risk of type 2 diabetes in healthy mainland China residents. EPMA J (2019) 10(3):227–37. doi: 10.1007/s13167-019-00181-2 PMC6695459 31462940

[5] Golubnitschaja O Costigliola V. General report & recommendations in predictive, preventive and personalised medicine 2012: White paper of the European association for predictive, preventive and personalised medicine. EPMA J (2012) 3(1):14. doi: 10.1186/1878-5085-3-14 23116135 PMC3485619

[6] Correa MM Thume E De Oliveira ER Tomasi E. Performance of the waist-to-height ratio in identifying obesity and predicting non-communicable diseases in the elderly population: A systematic literature review. Arch Gerontol Geriatr (2016) 65:174–82. doi: 10.1016/j.archger.2016.03.021 27061665

[7] Ashwell M Gunn P Gibson S. Waist-to-height ratio is a better screening tool than waist circumference and BMI for adult cardiometabolic risk factors: Systematic review and meta-analysis. Obes Rev (2012) 13(3):275–86. doi: 10.1111/j.1467-789X.2011.00952.x 22106927

[8] Chen Z Hu H Chen M Luo X Yao W Liang Q. Association of triglyceride to high-density lipoprotein cholesterol ratio and incident of diabetes mellitus: A secondary retrospective analysis based on a Chinese cohort study. Lipids Health Dis (2020) 19(1):33. doi: 10.1186/s12944-020-01213-x 32131838 PMC7057518

[9] Lim TK Lee HS Lee YJ. Triglyceride to HDL-cholesterol ratio and the incidence risk of type 2 diabetes in community dwelling adults: A longitudinal 12-year analysis of the Korean genome and epidemiology study. Diabetes Res Clin Pract (2020) 163:108150. doi: 10.1016/j.diabres.2020.108150 32305400

[10] Zheng D Li H Ai F Sun F Singh M Cao X. Association between the triglyceride to high-density lipoprotein cholesterol ratio and the risk of type 2 diabetes mellitus among Chinese elderly: The Beijing longitudinal study of aging. BMJ Open Diabetes Res Care (2020) 8(1):e000811. doi: 10.1136/bmjdrc-2019-000811 PMC7206911 32205325

[11] Wakabayashi I Daimon T. The "cardiometabolic index" as a new marker determined by adiposity and blood lipids for discrimination of diabetes mellitus. Clin Chim Acta (2015) 438:274–8. doi: 10.1016/j.cca.2014.08.042 25199852

[12] Higashiyama A Wakabayashi I Okamura T Kokubo Y Watanabe M Takegami M. The risk of fasting triglycerides and its related indices for ischemic cardiovascular diseases in Japanese community dwellers: the suita study. J Atheroscler Thromb (2021) 28(12):1275–88. doi: 10.5551/jat.62730 PMC8629703 34053965

[13] Qiu Y Yi Q Li S Sun W Ren Z Shen Y. Transition of cardiometabolic status and the risk of type 2 diabetes mellitus among middle-aged and older Chinese: A national cohort study. J Diabetes Investig (2022) 13(8):1426–37. doi: 10.1111/jdi.13805 PMC9340876 35426487

[14] Acosta-Garcia E Concepcion-Paez M. [Cardiometabolic index as a predictor of cardiovascular risk factors in adolescents]. Rev Salud Publica (Bogota) (2018) 20(3):340–5. doi: 10.15446/rsap.V20n3.61259 30844007

[15] Liu X Wu Q Yan G Duan J Chen Z Yang P. Cardiometabolic index: a new tool for screening the metabolically obese normal weight phenotype. J Endocrinol Invest (2021) 44(6):1253–61. doi: 10.1007/s40618-020-01417-z 32909175

[16] Wang Z He S Chen X. Capacity of different anthropometric measures to predict diabetes in a Chinese population in southwest China: A 15-year prospective study. Diabetes Med (2019) 36(10):1261–7. doi: 10.1111/dme.14055 31215075

[17] Liu Y Wang W. Sex-specific contribution of lipid accumulation product and cardiometabolic index in the identification of nonalcoholic fatty liver disease among Chinese adults. Lipids Health Dis (2022) 21(1):8. doi: 10.1186/s12944-021-01617-3 35027066 PMC8759215

[18] Zuo YQ Gao ZH Yin YL Yang X Feng PY. Association between the cardiometabolic index and hyperuricemia in an asymptomatic population with normal body mass index. Int J Gen Med (2021) 14:8603–10. doi: 10.2147/IJGM.S340595 PMC8627282 34849005

[19] Li FE Luo Y Zhang FL Zhang P Liu D Ta S. Association between cardiometabolic index and stroke: A population- based cross-sectional study. Curr Neurovasc Res (2021) 18(3):324–32. doi: 10.2174/1567202618666211013123557 34645376

[20] Wang HY Shi WR Yi X Wang SZ Luan SY Sun YX. Value of reduced glomerular filtration rate assessment with cardiometabolic index: insights from a population-based Chinese cohort. BMC Nephrol (2018) 19(1):294. doi: 10.1186/s12882-018-1098-8 30359237 PMC6202850

[21] Okamura T Hashimoto Y Hamaguchi M Obora A Kojima T Fukui M. Ectopic fat obesity presents the greatest risk for incident type 2 diabetes: a population-based longitudinal study. Int J Obes (Lond) (2019) 43(1):139–48. doi: 10.1038/s41366-018-0076-3 29717276

[22] American Diabetes Association. 2. Classification and diagnosis of diabetes: Standards of medical care in diabetes-2021. Diabetes Care (2021) 44(Suppl 1):S15–33. doi: 10.2337/dc21-S002 33298413

[23] Robson ME Tung N Conte P Im SA Senkus E Xu B. OlympiAD final overall survival and tolerability results: Olaparib versus chemotherapy treatment of physician's choice in patients with a germline BRCA mutation and HER2-negative metastatic breast cancer. Ann Oncol (2019) 30(4):558–66. doi: 10.1093/annonc/mdz012 PMC6503629 30689707

[24] Skrivankova VW Richmond RC Woolf B Davies NM Swanson SA VanderWeele TJ. Strengthening the reporting of observational studies in epidemiology using mendelian randomisation (STROBE-MR): explanation and elaboration. BMJ (2021) 375:n2233. doi: 10.1136/bmj.n2233 34702754 PMC8546498

[25] Zhu F Chen C Zhang Y Chen S Huang X Li J. Elevated blood mercury level has a non-linear association with infertility in U.S. women: Data from the NHANES 2013-2016. Reprod Toxicol (2020) 91:53–8. doi: 10.1016/j.reprotox.2019.11.005 31756438

[26] Haneuse S VanderWeele TJ Arterburn D. Using the e-value to assess the potential effect of unmeasured confounding in observational studies. JAMA (2019) 321(6):602–3. doi: 10.1001/jama.2018.21554 30676631

[27] von Elm E Altman DG Egger M Pocock SJ Gotzsche PC Vandenbroucke JP. The strengthening the reporting of observational studies in epidemiology (STROBE) statement: guidelines for reporting observational studies. Int J Surg (2014) 12(12):1495–9. doi: 10.1016/j.ijsu.2014.07.013 25046131

[28] Wang H Chen Y Sun G Jia P Qian H Sun Y. Validity of cardiometabolic index, lipid accumulation product, and body adiposity index in predicting the risk of hypertension in Chinese population. Postgrad Med (2018) 130(3):325–33. doi: 10.1080/00325481.2018.1444901 29478365

[29] Shi WR Wang HY Chen S Guo XF Li Z Sun YX. Estimate of prevalent diabetes from cardiometabolic index in general Chinese population: a community-based study. Lipids Health Dis (2018) 17(1):236. doi: 10.1186/s12944-018-0886-2 30314516 PMC6186046

[30] Kahn SE Hull RL Utzschneider KM. Mechanisms linking obesity to insulin resistance and type 2 diabetes. Nature (2006) 444(7121):840–6. doi: 10.1038/nature05482 17167471

[31] Barazzoni R Gortan CG Ragni M Nisoli E. Insulin resistance in obesity: an overview of fundamental alterations. Eat Weight Disord (2018) 23(2):149–57. doi: 10.1007/s40519-018-0481-6 29397563

[32] Sharma AM Lau DC. Obesity and type 2 diabetes mellitus. Can J Diabetes (2013) 37:63–4. doi: 10.1016/j.jcjd.2013.03.360 24070794

[33] Goodpaster BH Kelley DE. Skeletal muscle triglyceride: marker or mediator of obesity-induced insulin resistance in type 2 diabetes mellitus? Curr Diabetes Rep (2002) 2(3):216–22. doi: 10.1007/s11892-002-0086-2 12643176

