# Supplementary material for: The nonlinear correlation between the cardiometabolic index and the risk of diabetes: A retrospective Japanese cohort study
Source: Front Endocrinol (Lausanne). 2023 Feb 16;14:1120277. doi: 10.3389/fendo.2023.1120277 (PMC9980900; doi:10.3389/fendo.2023.1120277)
Supplement: Supplementary file 1 [file Table_1.docx]

**Table S1** The result of the two-piecewise linear regression model

| Incident DM | HR (95%CI), | P |
| --- | --- | --- |
| Fitting model by standard linear regression | 1.65 (1.43, 1.90) | <0.0001 |
| Fitting model by two-piecewise linear regression | |  |
| Inflection point of CMI | 1.01 |  |
| ≤1.01 | 2.96 (1.96, 4.46) | <0.0001 |
| >1.01 | 1.27 (0.98, 1.64) | 0.0702 |
| P for the log-likelihood ratio test | 0.003 |  |

We adjusted for gender, age, ethanol consumption, smoking status, habit of exercise, SBP, DBP, ALT, AST, GGT, TC, HbA1c, and FPG.

HR: hazard ratios; CI: confidence; DM: diabetes mellitus; CMI: cardiometabolic index

**Table S2** Variables included age, gender, BMI, habit of exercise, smoking status and ethanol consumption interacted with CMI

| Characteristic |  | P-value |
| --- | --- | --- |
| Age |  | 0.220 |
| Gender |  | **<0.001** |
| BMI |  | **<0.001** |
| Habit of exercise |  | **0.005** |
| Smoking status |  | **0.037** |
| Ethanol consumption |  | 0.608 |

Note 1: P>0.05 suggested that there was no interaction

Note 2: Above model was adjusted for gender, age, ethanol consumption, smoking status, habit of exercise, SBP, DBP, ALT, AST, GGT, TC, HbA1c, and FPG.

Note 3: In each case, the model is not adjusted for the stratification variable
